# Nanoparticle-Based Therapies for Cardiovascular Diseases: A Literature Review of Recent Advances and Clinical Potential

**DOI:** 10.7759/cureus.72808

**Published:** 2024-10-31

**Authors:** Gaurav Jha, Ritika B Sharma, Sruthi Sridhar, Disha Hayagreev, Tanya Sinha, Harsimran Kaur, Adrija Das, Reddy Lahari Bollineni

**Affiliations:** 1 Trauma and Orthopaedics, Leicester Royal Infirmary, Leicester, GBR; 2 Geriatrics, Pinderfields General Hospital, MidYorkshire, GBR; 3 Emergency Department, Croydon Health Services NHS Trust, London, GBR; 4 Emergency Department, Basingstoke and North Hampshire Hospital, Basingstoke, GBR; 5 Emergency Medicine, South Tyneside and Sunderland NHS Foundation Trust, South Sheilds, GBR; 6 Accident and Emergency, Ealing Hospital, Southall, GBR; 7 Medicine, Newcastle University, Newcastle, GBR; 8 Internal Medicine, Royal Stoke Hospital, Stoke-on-Trent, GBR

**Keywords:** cardiovascular diseases, cardiovascular intervention, nanoparticles, new drug delivery systems, novel treatment, targeted therapy, therapeutic interventions

## Abstract

Cardiovascular diseases (CVDs) present a significant global health burden and remain the leading cause of morbidity and mortality worldwide. Conventional pharmacological therapies have yielded limited success in addressing the underlying pathophysiology of these diseases, leading to the exploration of novel therapeutic approaches. Nanotechnology is transforming cardiovascular disease management by enabling the engineering of materials at the atomic and molecular levels. This has led to the development of advanced diagnostic tools with unparalleled accuracy and sensitivity in detecting these diseases. By enabling targeted drug delivery, enhancing imaging techniques, and facilitating personalized therapies, nanotechnology promises significant advancements in the diagnosis, treatment, and prevention of cardiovascular diseases. This narrative review provides a comprehensive outlook on the recent advancements in nanoparticle-based therapies for cardiovascular diseases. We delve into the diverse applications of various nanoparticle types, exploring their potential to surpass the limitations of conventional treatments and improve clinical outcomes. Additionally, we critically examine the challenges and future directions of this rapidly evolving field, emphasizing the need for rigorous clinical evaluation.

## Introduction and background

Cardiovascular diseases (CVDs) continue to be the leading cause of mortality worldwide, accounting for an estimated 17.9 million deaths annually, which represents about 32% of all global deaths [1]. This group of disorders, including coronary artery disease, heart failure, and stroke, poses a significant burden on healthcare systems and societies worldwide [2]. Despite considerable advancements in conventional therapeutic strategies, such as pharmacological interventions and surgical procedures, the complexity of CVDs presents ongoing challenges, particularly due to limitations that hinder their effectiveness in managing CVDs [3].

Traditional pharmacological strategies in CVD treatments frequently encounter pharmacokinetic limitations such as poor drug bioavailability, systemic side effects, limited biocompatibility, inadequate targeting capabilities, and suboptimal drug release profiles. Despite advancements in the last decade, pharmacotherapy's therapeutic efficacy remains insufficient due to factors like non-specific cytotoxicity, poor solubility and absorption, first-pass metabolism, and low bioavailability of conventional cardiovascular drugs [4,5]. These issues often lead to reduced therapeutic efficacy, requiring the exploration of alternative approaches that can overcome these hurdles and potentially improve patient outcomes. In this context, nanotechnology has emerged as a promising field with the potential to revolutionize cardiovascular medicine [6].

Nanotechnology, involving the manipulation of materials at the nanoscale (1-100 nm), has emerged as a promising frontier in cardiovascular medicine, particularly in drug delivery systems. Nanoparticles (NPs), characterized by their small size, high surface area-to-volume ratio, and customizable surface properties, offer potential solutions to the limitations of conventional CVD therapies [7,8]. These unique properties and due to their unique physicochemical properties, offer several key advantages in cardiovascular drug delivery, such as improved solubility, enhanced bioavailability, controlled release kinetics, and targeted delivery. These properties help in overcoming the limitations of conventional drug formulations, ensuring that therapeutic agents are delivered more efficiently and with fewer side effects. Nanoparticles can be engineered to facilitate precise localization and release of drugs, reducing systemic toxicity and enhancing therapeutic outcomes [9,10]

**Figure 1:**
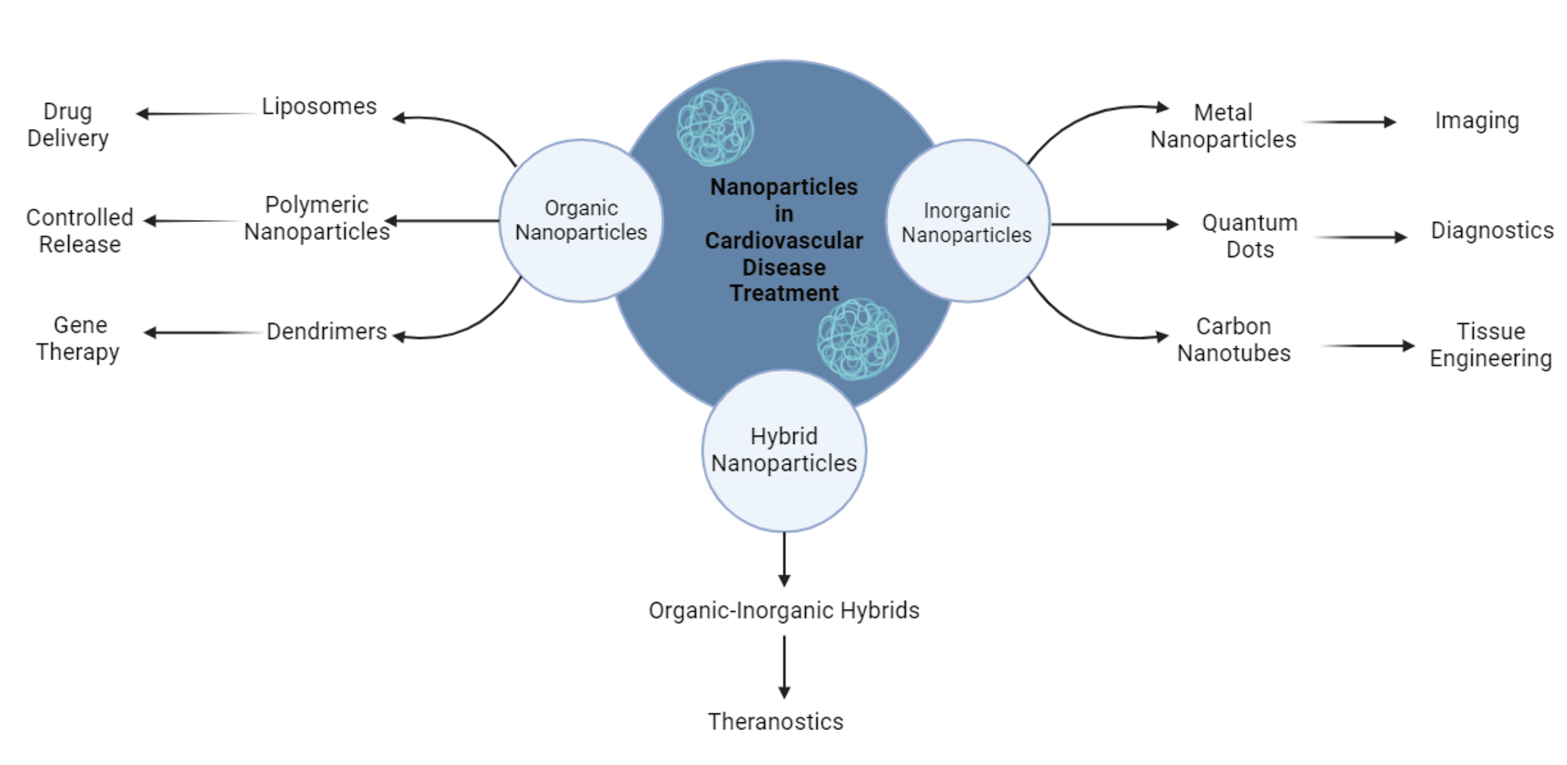
Classification and Applications of Nanoparticles in Cardiovascular Disease Treatment

Different types of NPs, such as liposomes, polymeric nanoparticles like poly(lactic-*co*-glycolic acid) (PLGA), dendrimers, inorganic metallic nanoparticles (e.g., gold and iron oxide), and hybrid systems like lipid-polymer hybrids, have been extensively explored for cardiovascular applications as shown in Table 1. These nanosystems have shown potential as versatile tools in cardiovascular medicine due to their capabilities for targeted drug delivery, controlled release, and simultaneous therapeutic and diagnostic functions, making them highly promising for advancing cardiovascular treatment strategies as depicted in Figure 1 [9-11]. Liposomes, with their phospholipid bilayers, can encapsulate diverse drug types, while polymeric nanoparticles offer precise release properties for sustained therapy. Dendrimers, owing to their branched structure, provide multiple sites for drug conjugation, and metallic nanoparticles contribute to both therapy and imaging applications [12]. 

These systems have been successfully used in cardiovascular disease management, targeting atherosclerotic plaques with anti-inflammatory drugs, enhancing thrombolysis with reduced bleeding risk, and improving cardiac repair after myocardial infarction. In cases of restenosis, nanoparticles enable targeted delivery of antiproliferative agents to prevent arterial narrowing post-angioplasty [10,12]. Despite these advancements, challenges such as optimizing biodistribution, ensuring long-term safety, and navigating regulatory complexities remain before their widespread clinical adoption [9,10,12].

## Review

**Figure 2:**
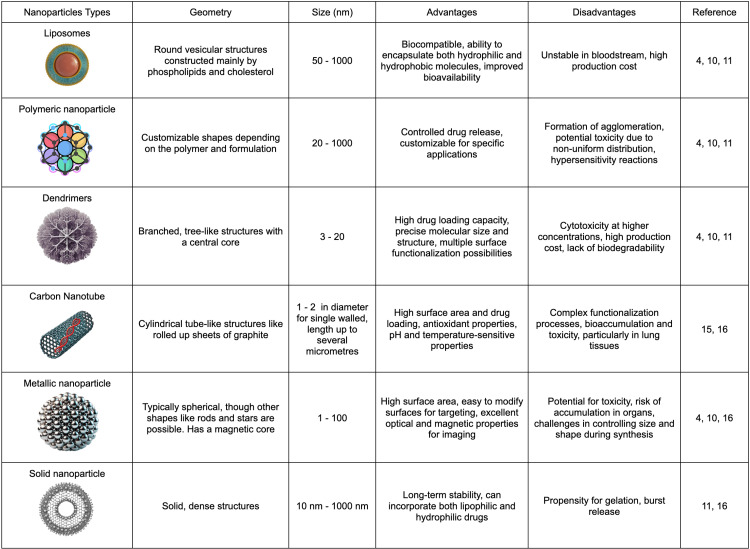
Comparison of Nanoparticle Types for Drug Delivery and Biomedical Applications

Nanoparticles offer a versatile platform for addressing various aspects of cardiovascular diseases, from atherosclerosis and thrombosis to myocardial infarction and heart failure. Their applications span a wide range of therapeutic strategies, including targeted drug delivery, gene therapy, and theranostic approaches combining diagnosis and treatment [13]. Liposomes, polymeric nanoparticles, dendrimers, and inorganic nanoparticles each possess unique characteristics (Figure 2) that make them suitable for specific cardiovascular applications. These nanoparticles can be engineered to target specific components of the cardiovascular system, such as endothelial cells, macrophages, or thrombi, enhancing the precision and efficacy of treatments [14,15]. They can carry a variety of therapeutic agents, including small molecule drugs, proteins, nucleic acids, and imaging contrast agents, often in combinations that would be impossible with conventional drug formulations [4, 16]. The following sections will delve into each type of nanoparticle, examining their specific applications, mechanisms of action, and potential impact on cardiovascular disease management.

Liposomes

Liposomes are one of the earliest and most well-researched nanoparticle systems for drug delivery, especially in the treatment of CVDs. These spherical vesicles, made of phospholipid bilayers, can encapsulate both hydrophilic and hydrophobic drugs, improving drug stability and bioavailability. Their structure mimics natural cellular membranes, providing excellent biocompatibility, and when modified with polyethylene glycol (PEG) to form "stealth" liposomes, they evade the immune system, extending their circulation time [17,18].

Mechanisms and Advantages of Liposomal Drug Delivery

Liposomes' ability to fuse with cell membranes allows for efficient intracellular drug delivery, particularly relevant in cardiovascular applications. PEGylation enhances their stealth properties, preventing rapid clearance by the reticuloendothelial system and improving their drug delivery efficacy [18,19]. This technology has been instrumental in increasing the bioavailability of various cardiovascular therapeutics.

Applications in Cardiovascular Diseases

*Atherosclerosis*: Liposomal formulations of statins, such as atorvastatin, have demonstrated superior effectiveness compared to free drug formulations in the treatment of atherosclerosis. In studies involving animal models, particularly ApoE-deficient mice, liposomal atorvastatin significantly reduced inflammation and stabilized atherosclerotic plaques more effectively than its free counterpart [20]. Research has shown that these liposomal formulations can decrease lipid accumulation in macrophages and reduce the levels of inflammatory markers, with liposomal atorvastatin outperforming free atorvastatin in plaque stabilization and anti-inflammatory effects [20,21].

*Thrombosis*: Nanoparticles have been explored for enhanced delivery of thrombolytic agents. Korin et al. developed shear-activated nanotherapeutics that specifically target obstructed blood vessels. In a mouse arterial thrombosis model, these nanoparticles resulted in rapid clot dissolution and restored blood flow with a markedly lower dose than free tissue plasminogen activator (tPA), potentially reducing bleeding risks [22].

*Myocardial infarction*: Nanoparticle-based delivery systems have demonstrated potential in reducing infarct size and improving cardiac function post-myocardial infarction. Fan et al. [23] developed a cardiac-targeting peptide-modified liposomal system for the co-delivery of vascular endothelial growth factor (VEGF) and hepatocyte growth factor (HGF). In a rat myocardial infarction model, this nanoparticle system significantly improved cardiac function and reduced infarct size compared to free growth factors [24].

Polymeric nanoparticles

Polymeric nanoparticles (PNPs) have garnered significant attention in the field of nanomedicine due to their versatile properties and potential for efficient bodily absorption [25]. Polymeric nanoparticles encompass a diverse array of systems, including solid nanoparticles, amphiphilic nanoparticles, dendrimers, and star-shaped structures, each with its own unique architectural features and characteristics [26]. Several non-biodegradable polymers, including polymethylmethacrylate (PMMA), poly(acrylamide), poly(styrene), and poly(acrylates), have been widely studied for various applications; however, these materials have been associated with chronic toxicity in long-term use, prompting a shift in focus toward biodegradable alternatives [27]. 

The most commonly used polymers for biodegradable nanocarriers include poly-lactic-*co*-glycolic acid (PLGA), polyglycolic acid (PGA), and polylactic acid (PLA). These materials are biodegradable because they can be naturally broken down into carbon dioxide and water, making them safe for drug delivery. Among these, PLGA is the most extensively studied for cardiovascular disease treatments due to its effective drug release and biocompatibility [11].

Mechanism and Advantages

PNPs are a versatile platform for drug delivery, capable of encapsulating drugs within their core or adsorbing them onto their surface. These nanoparticles can be engineered for a controlled drug release through mechanisms such as diffusion, erosion, and stimuli-responsive systems (e.g., pH, temperature, light). Stimuli-responsive PNPs can release drugs in response to specific internal or external triggers, enhancing the precision of drug delivery [28,29]. The functionalization of PNPs with targeting ligands (e.g., enzymes, antibodies, peptides) can enhance site-specific drug delivery in specific cardiovascular tissues and types, improving therapeutic outcomes and reducing side effects [28,30].

Applications in Cardiovascular Diseases

*Restenosis prevention*: PLGA nanoparticles loaded with antiproliferative agents like paclitaxel have shown significant efficacy in reducing restenosis rates following angioplasty (delivered via catheter or balloon angioplasty) by inhibiting smooth muscle cell proliferation and promoting endothelial regeneration. A study by Westedt et al. demonstrated that paclitaxel-loaded PLGA nanoparticles to stented arteries in rabbits resulted in a 50% reduction in neointimal area compared to non-targeted nanoparticles with control segments [31]. 

*Gene therapy for myocardial infarction*: Gene therapy using PNPs or cationic microbubbles to deliver siRNA or shRNA against PHD2 can improve angiogenesis, reduce infarct size, and enhance cardiac function post-myocardial infarction. PNPs such as PLGA, to deliver siRNA or shRNA targeting prolyl hydroxylase domain protein 2 (PHD2) have demonstrated substantial potential for cardiac regeneration after myocardial infarction (MI). By inhibiting PHD2, the pro-angiogenic HIF-1α pathway is stabilized, leading to enhanced angiogenesis and a notable 24% reduction in infarct size in a mouse model [32,33]. 

*Anti-inflammatory therapy*: Polymeric nanoparticles have been employed to deliver anti-inflammatory agents to reduce vascular inflammation in atherosclerosis. A study by Koga et al. showed that PLGA nanoparticle-mediated delivery of pitavastatin has been shown to prevent inflammation and ameliorate features associated with plaque ruptures in hyperlipidemic mice [34,35].

Dendrimer-based therapies

Dendrimers are highly branched, tree-like polymers that offer a unique structure with multiple surface functional groups, making them ideal for drug conjugation and targeted delivery. Their architecture provides several advantages, including a high drug-loading capacity, multifunctionality, and precise control over their size, shape, and surface properties. These features allow dendrimers to enhance the solubility and bioavailability of drugs, making them highly effective in various therapeutic applications, including cancer and cardiovascular treatments [36].

Mechanism and Advantages

Dendrimers can transport drugs either by encapsulating them within their internal cavities or by conjugating them to their numerous surface groups with some ligands or antibodies for active targeting. Their highly branched structure allows for a high drug-loading capacity, while their well-defined size and shape contribute to predictable and consistent pharmacokinetic profiles. Additionally, the multivalent surface of dendrimers can be functionalized with targeting ligands or imaging agents, which makes them ideal candidates for theranostic applications, combining therapy with diagnostics in one platform. Polyamidoamine (PAMAM) dendrimer is the most frequent selection among the numerous dendrimers used for drug delivery [7, 36,37].

Applications in Cardiovascular Diseases

*Antioxidant therapy*: Dendrimers have been used to deliver antioxidants for the treatment of oxidative stress-related cardiovascular conditions. For example, Deng et al. study indicates that lipoic acid (LA) reduces infarct size and preserves cardiac function in rats with myocardial ischemia-reperfusion injury by activating the PI3K/Akt pathway and inducing cytoprotective genes when compared to the free form of the oxidant [38]. 

*Anti-inflammatory therapy*: Dendrimer-based delivery of anti-inflammatory agents significantly enhances their efficacy, stability, and targeted intracellular release, thereby improving treatment outcomes for vascular inflammation. A study by Ficker et al. used pyrrolidone-modified PAMAM dendrimers to deliver indomethacin showed improved anti-inflammatory properties and reduced toxicity profile, making them a potential treatment for atherosclerosis and cardiovascular disease compared to free indomethacin [39].

*Gene therapy*: Dendrimers have been explored for the delivery of nucleic acids in cardiovascular applications. Studies suggest that dendrimers are effective, non-toxic, and efficient carriers for delivering nucleic acids in cardiovascular gene therapy applications. Studies have suggested that PAMAM dendrimers modified with arginine can effectively deliver microRNA-126 (miR-126) mimics to promote angiogenesis post-MI with high transfection efficiency and low cytotoxicity. In a rat model, this dendrimer-based delivery system improved cardiac function and reduced infarct size by 28% compared to control treatments [40].

Inorganic nanoparticles

Inorganic nanoparticles are a diverse group of nanomaterials that offer unique physicochemical properties, making them highly suitable for biomedical applications, including the treatment of CVDs. These nanoparticles are generally categorized based on their composition and include metallic, non-metallic, carbon-based nanomaterials, and quantum dots, each with specific properties that make them advantageous for particular therapeutic and diagnostic roles in medicine [41].

Carbon-based nanoparticles, like carbon nanotubes (CNTs) and graphene, are valuable in cardiovascular applications due to their structural and electrical properties. CNTs can deliver genetic materials such as RNA or DNA directly into cells, making them suitable for gene therapy. Graphene and graphene oxide excel in biosensors and drug delivery systems, aiding in diagnostics and targeted therapies. Quantum dots (QDs), known for their fluorescence, are useful for imaging in cardiovascular research, allowing real-time tracking of drug delivery and cellular responses. These materials enhance both therapeutic and diagnostic approaches in cardiovascular medicine [42,43].

Mechanism and Advantages

Inorganic nanoparticles, such as gold, iron oxide, and silica nanoparticles, possess distinctive optical, magnetic, and thermal characteristics that make them well-suited for a broad range of cardiovascular medicine applications. These properties can be leveraged for imaging purposes, where nanoparticles like gold and iron oxide enhance contrast in modalities such as computed tomography and magnetic resonance imaging. In drug delivery, their surfaces can be easily functionalized with therapeutic agents or targeting ligands, enabling the targeted administration of drugs to diseased tissues while minimizing systemic exposure. Additionally, these nanomaterials exhibit notable antimicrobial properties, rendering them effective as bactericidal, fungicidal, and antiviral agents [7,10,44].

Applications in Cardiovascular Diseases

*Photothermal therapy*: Gold nanoparticles (AuNP) have demonstrated remarkable efficacy in the photothermal ablation of atherosclerotic plaques. A study conducted by Kharlamov et al. revealed that near-infrared photothermal therapy led to substantial degradation of atherosclerotic lesions, with a 44.8% reduction observed in the silica-gold nanoparticle group and a 43.2% reduction in the gold iron-bearing nanoparticle group. In contrast, traditional therapies such as statin drugs or stent placement under anti-platelet protection resulted in only a 0.5-12.7% reduction in atherosclerotic lesions [45]. Furthermore, another study found that gold nanoparticles can be used to deliver anti-inflammatory drugs directly to targeted sites within the cardiovascular system, effectively reducing inflammation and improving therapeutic outcomes [46].

*Targeted drug delivery*: Recent studies have highlighted the emerging therapeutic potential of iron oxide nanoparticles in cardiovascular medicine. Xiong et al. reported that inorganic nanoparticles (IONPs) demonstrated superior cardioprotective effects, including reducing infarct size, improving left ventricular developed pressure recovery after ischemia-reperfusion, enhancing cell viability, and lowering reactive oxygen species levels. These findings suggest that IONPs may offer advantages over conventional therapies in terms of both efficacy and dosage requirements [47].

*Molecular imaging*: Inorganic nanoparticles have shown great potential as contrast agents for cardiovascular imaging. Investigations by Liu et al. have demonstrated the promise of inorganic nanoparticles, specifically iron oxide-based PLGA nanoparticles, as contrast agents for cardiovascular imaging. These nanoparticles exhibited superior performance compared to their non-targeted counterparts in terms of imaging contrast and targeting accuracy for thrombus sites, highlighting their enhanced efficacy for molecular imaging of thrombosis [48].

Challenges and future directions

Despite promising results from preclinical studies, several challenges hinder the clinical translation of nanoparticle-based therapies in cardiovascular medicine. Inorganic nanoparticles often face issues such as potential long-term toxicity, accumulation in non-target tissues, and the need for comprehensive evaluations of their biodistribution and clearance [41-43]. Dendrimer-based therapies encounter difficulties like cytotoxicity at higher doses, costly synthesis, and lack of biodegradability, which complicate their clinical adoption [38-40]. Polymeric nanoparticles require further optimization of drug loading, release kinetics, and stability while addressing concerns about toxicity from degradation [25, 26]. Liposomes present challenges related to scalable production, stability during storage, and risks of immune reactions with repeated dosing, alongside difficulties in achieving precise targeting in cardiovascular tissues [18,19].

Biological challenges include ensuring biocompatibility, as long-term safety profiles are often unclear, raising concerns about accumulation in non-target organs and immune responses [49]. Optimizing biodistribution and clearance is crucial, as nanoparticle size, shape, and surface properties significantly influence their behavior in vivo. Achieving efficient targeting in the complex cardiovascular environment remains a hurdle, despite promising in vitro results [9-11, 50]. Technological challenges involve scaling up production to meet GMP standards, maintaining formulation stability during storage, and optimizing drug release kinetics [51, 52]. Regulatory frameworks remain a significant hurdle as nanoparticle-based therapies are often classified as combination products, creating ambiguity in approval pathways. Addressing these challenges requires collaborative efforts to establish guidelines that accommodate the unique properties of nanoparticles. Additionally, the lack of standardized protocols for evaluating nanoparticle safety and efficacy necessitates the development of new assessment methods [53].

The field of nanoparticle-based therapies for cardiovascular diseases is advancing through key innovations. Smart, stimuli-responsive nanoparticles can selectively deliver drugs, improving targeting. Biomimetic approaches, which involve creating particles that mimic natural biological structures like cell membranes, improve biocompatibility and reduce immunogenicity. Combination therapies enable the co-delivery of drugs, addressing complex disease pathways synergistically. Integrating therapeutic and diagnostic functions allows real-time monitoring, supporting personalized approaches [4,7,9,10,37]. Advances in manufacturing, particularly through microfluidics and 3D printing, provide precise control (size, shape, and composition) over nanoparticle properties and enable scalable production. Currently, the absence of standardized safety and efficacy assessment protocols limits the progression of nanoparticle-based therapies from bench to bedside. Establishing universally accepted metrics and protocols for evaluating long-term safety, biodegradability, and therapeutic outcomes is crucial for advancing clinical use. Collaborative efforts between researchers, industry, and regulators are essential for developing standardized protocols, facilitating clinical translation. Together, these advancements hold promise for overcoming current challenges and bringing nanoparticle-modulated therapies closer to clinical use [54, 55].

## Conclusions

Nanoparticle-based therapies hold a significant promise for revolutionizing cardiovascular disease treatment by improving drug delivery, minimizing side effects, and enhancing therapeutic outcomes. Platforms like liposomes, polymeric nanoparticles, dendrimers, and inorganic nanoparticles offer versatile solutions for the complexities of cardiovascular medicine. Although preclinical data show improved targeting and novel therapies, translating these advances into clinical practice faces challenges, including biocompatibility, scalable production, and regulatory hurdles. The future lies in developing multifunctional nanoparticles that can adapt to the cardiovascular environment, deliver combination therapies, and integrate diagnostics with treatment. As the understanding of nanoparticle interactions and manufacturing techniques advances, more therapies will likely progress to clinical trials. Success will require continued collaboration between researchers, clinicians, and regulatory bodies. With strategic efforts, nanoparticle-based therapies could significantly enhance the management of cardiovascular diseases, offering better patient outcomes in this critical field.
